# Comorbidity profiling identifies potential subtype of elderly patients with nasopharyngeal carcinoma

**DOI:** 10.1093/oncolo/oyae063

**Published:** 2024-04-16

**Authors:** Ying Li, Yuhui Pan, Zongwei Huang, Lishui Wu, Wenxi Wu, Siqi Xu, Zihan Chen, Xin Chen, Jun Lu, Sufang Qiu

**Affiliations:** Clinical Oncology School of Fujian Medical University, Fujian Cancer Hospital, Fujian, People’s Republic of China; Clinical Oncology School of Fujian Medical University, Fujian Cancer Hospital, Fujian, People’s Republic of China; Clinical Oncology School of Fujian Medical University, Fujian Cancer Hospital, Fujian, People’s Republic of China; Clinical Oncology School of Fujian Medical University, Fujian Cancer Hospital, Fujian, People’s Republic of China; Clinical Oncology School of Fujian Medical University, Fujian Cancer Hospital, Fujian, People’s Republic of China; Clinical Oncology School of Fujian Medical University, Fujian Cancer Hospital, Fujian, People’s Republic of China; Clinical Oncology School of Fujian Medical University, Fujian Cancer Hospital, Fujian, People’s Republic of China; Clinical Oncology School of Fujian Medical University, Fujian Cancer Hospital, Fujian, People’s Republic of China; Clinical Oncology School of Fujian Medical University, Fujian Cancer Hospital, Fujian, People’s Republic of China; Clinical Oncology School of Fujian Medical University, Fujian Cancer Hospital, Fujian, People’s Republic of China; Fujian Key Laboratory of Translational Cancer Medicine, Fujian, People’s Republic of China; Fujian Provincial Key Laboratory of Tumor Biotherapy, Fujian, People’s Republic of China

**Keywords:** nasopharyngeal carcinoma, elderly patients, comorbidity, all-cause mortality, prognosis

## Abstract

**Background:**

Few studies have assessed the comprehensive associations among comorbid diseases in elderly patients with nasopharyngeal carcinoma (NPC). This study sought to identify potential comorbidity patterns and explore the relationship of comorbidity patterns with the mortality risk in elderly patients with NPC.

**Methods:**

A total of 452 elderly patients with NPC were enrolled in the study. The network analysis and latent class analysis were applied to mine comorbidity patterns. Propensity score matching was used for adjusting confounders. A restricted cubic spline model was used to analyze the nonlinear association between age and the risk of all-cause mortality.

**Results:**

We identified 2 comorbidity patterns, metabolic disease-related comorbidity (MDRC) and organ disease-related comorbidity (ODRC) in elderly patients with NPC. Patients in MDRC showed a significantly higher risk of all-cause mortality (71.41% vs 87.97%, HR 1.819 [95% CI, 1.106-2.994], *P* = .031) and locoregional relapse (68.73% vs 80.88%, HR 1.689 [95% CI, 1.055-2.704], *P* = .042). Moreover, in patients with MDRC pattern, we observed an intriguing inverted S-shaped relationship between age and all-cause mortality among patients aged 68 years and older. The risk of mortality up perpetually with age increasing in ODRC group, specifically within the age range of 68-77 years (HR 4.371, 1.958-9.757).

**Conclusion:**

Our study shed light on the potential comorbidity patterns in elderly patients with NPC, thereby providing valuable insights into the development of comprehensive health management strategies for this specific population.

## Teaser

Comorbidity is a prognostic factor in the assessment of elderly patients with nasopharyngeal carcinoma. This study analyzed the comorbidities observed in elderly patients, looking for insights into comprehensive health management strategies specifically tailored to this particular population.

**Implications for Practice**

Comorbidity emerges as a crucial prognostic factor in the assessment of elderly patients with nasopharyngeal carcinoma (NPC). The researchers analyzed the comorbidities observed in each elderly NPC individuals at baseline and then identified 2 potential comorbidity patterns, namely metabolic disease-related comorbidity and organ disease-related comorbidity. The findings may offer valuable insights into the formulation of comprehensive health management strategies, specifically tailored to this particular population.

## Introduction

As living standards improve and medical technology advances, there has been a gradual extension of life expectancy, leading to a notable growth in the proportion of the elderly population.^1^ This demographic shift poses significant challenges in many fields, among which, the prevalent increase of chronic diseases accompanied by aging is a prominent concern.^2^ It has shown that comorbid chronic diseases affect elderly patients with an increasing frequency, particularly the impact of comorbidities on cancer risk and survivorship in patients with tumor.^3^ Comorbidity among patients with cancer refers to the presence of additional coexisting disorders or chronic conditions when patients labeled with a cancer diagnosis.^4^ As it stands, it is estimated that at least one comorbidity is present in 75% of patients with cancer.^5^ They negatively and disproportionately affect underserved populations and influence cancer diagnosis, progression, and treatment selection.^3,6^

Nasopharyngeal carcinoma (NPC), a malignant tumor originating from the nasopharynx epithelium, demonstrates the highest incidence rates in China.^7^ Unlike the bimodal distribution observed in low-risk populations, the relative risk of NPC exhibits an age-dependent pattern, reaching its peak around 55 years of age and subsequently declining in individuals aged over 60 years.^8^ Notably, data obtained from the Hong Kong Cancer Registry reveal that the proportion of new NPC cases in individuals aged 70 years and above ranges from 10.6% to 14.4%.^9^ Multiple retrospective studies have consistently highlighted the significantly poor survival outcomes experienced by elderly patients with NPC, with reported 5-year overall survival (OS) rates ranging from 43.9% to 61.8%.^10,11^ Given the diverse array of patient phenotypes encompassed within this age group, the management of NPC in elderly patients poses notable challenges. Factors such as delayed diagnosis, the presence of comorbidities, and suboptimal functional states further contribute to the complexity of managing this patient population.^7,12^

Comorbidity emerges as a crucial prognostic factor in the assessment of elderly patients with NPC.^13^ Studies have demonstrated that the presence of comorbidities in both endemic and non-endemic areas is significantly associated with worse OS.^13^ The incidence of comorbidity in elderly patients with NPC ranged from 22.4% to 58%, in which the variation observed in comorbidity rates could be attributed to the utilization of different assessment tools, such as the Charlson comorbidity index (CCI) and the Adult Comorbidity Evaluation-27 (ACE-27) instrument.^12-15^ However, it is noteworthy that these methods often lack specific information regarding individual comorbid diseases and fail to illuminate comprehensive associations among different medical conditions in the context of elderly patients with NPC.

Here, we focused on the prevalence and the aggregation of different comorbid diseases, and mined comorbidity patterns from potential disease associations by latent class analysis (LCA), providing valuable insights into current treatment practices and comprehensive management of comorbidity in elderly patients with NPC.

## Methods and materials

### Patients and clinicopathologic variables

Four hundred and fifty-two elderly patients with NPC (aged 65 years or older) treated in Fujian Cancer Hospital, from January 2015 to December 2021 were enrolled in this study. All patients were confirmed by pathological examinations and completed radical intensity-modulated radiotherapy (IMRT). All cases underwent restaging in accordance with the 8th edition American Joint Committee on Cancer by 2 radiologists. The exclusion criteria encompassed distant metastasis at initial diagnosis, prior antitumor treatment, and lost to follow-up. The study received ethical approval from the Ethics Committee of Fujian Cancer Hospital (K2022-203-01). No additional patient informed consent that was specific to this study was required given its retrospective nature.

Comorbidity was obtained through medical records collected by self-reported chronic diseases diagnosed previously, supplemented by the results of physical examination, equipment inspection, or blood test at baseline. Included covariates were age, sex, TNM stage, pretreatment plasma EBV DNA load, and treatment modality.

### Treatment and follow-up

All patients received IMRT, of which the detailed description has been described previously.^16^ Briefly, a total dose of 59.36-76.15 Gy to the planning target volumes of gross tumor volume at primary tumors, 48-74.25 Gy to the planning target volumes of gross tumor volume at positive lymph nodes in 30-38 fractions. The application of platinum-based induction chemotherapy (IC) was chosen at physicians’ discretion, taking into consideration the pretreatment features or tolerance of patients. The commonly used regimens were gemcitabine or paclitaxel plus platinum with 1-6 cycles every 3 weeks, besides docetaxel or 5-fuorouracil with platinum or other regimens. Platinum-based concurrent chemotherapy was given on days 1 and 22 of radiotherapy with 1-3 cycles. The utilization of adjuvant chemotherapy or targeted therapy was determined by the clinical judgment and tumor status of the physicians.

Post-treatment, regular follow-up assessments were conducted for all patients at 3-month intervals during the initial 2 years, followed by biannual evaluations over the subsequent 3 years, and annual assessments thereafter. The documentation of survival outcomes and tumor status relied upon the collection of clinical records and communication via telephone. In cases suspected with disease progression, efforts were made to confirm through pathological examination whenever feasible. For inaccessible lesions with typical radiographic characteristics, the diagnosis was based on a minimum of 2 imaging modalities, regardless of the presence or absence of clinical symptoms.

The principal endpoint of this study was OS, encompassing the duration from the completion of IMRT to mortality from any cause. The secondary endpoints included locoregional relapse-free survival (LRFS, the span until the identification of locoregional failure or death), distant metastasis-free survival (DMFS, the span until documented distant metastasis or death), and failure-free survival (the duration until locoregional failure, distant metastasis, or any-cause death). In the event of tumor progression, the decision to initiate salvage therapy was made collaboratively between the patients and physicians.

### Assessment of comorbidity patterns

In light of the unique characteristics pertaining to the population and environmental geographic factors influencing the disease spectrum, our study referred to chronic conditions among Chinese elderly from a previous China Health and Retirement Longitudinal Study, a comprehensive interdisciplinary research endeavor aimed at analyzing the aging phenomenon within China’s population.^17-19^ Diseases that were easily confused and misreported, such as chronic pneumonia as diagnosed by imaging, were excluded. Finally, we incorporated 10 diseases in this study, including: 1. hypertension; 2. dyslipidemia (hyperlipidemia or hypercholesterolemia); 3. diabetes; 4. coronary heart disease, angina, congestive heart failure, or other cardiac insufficiencies; 5. stroke; 6. chronic lung diseases, such as emphysema, pulmonary bulla, atelectasis, bronchiectasis, and tuberculosis; 7. stomach diseases (peptic ulcer or chronic gastritis); 8. chronic liver diseases (including cirrhosis or chronic hepatitis); 9. fatty liver; 10. malignant tumor. We dichotomized each of the included chronic diseases (yes/no).

Network analysis was performed to explore the interaction of comorbidity in elderly patients using Spearman’s rho test. Each chronic disease is visualized by a dot in the network analysis. The presence of concurrent chronic diseases in a patient is illustrated by the connection of corresponding dots through line segments, the thickness of which is contingent upon the frequency at which the 2 chronic diseases co-occur. A higher thickness signifies a higher risk of being diagnosed with a related disease.^20^ Comorbidity patterns were identified using LCA, a robust method characterized by its ability to identify distinct patient underlying subgroups through rigorous statistical inference from the maximum likelihood estimation approach considering multiple variables concurrently without considering the outcome.^21^ The LCA was performed using the “poLCA” R package.^22^ To determine the optimal number of classes, models ranging from 2 to 5 clusters were assessed for model fitting using Akaike information criterion (AIC), Bayesian information criterion (BIC), maximum likelihood ratio test, and chi-square goodness-to-fit test.^23^ Each case would be allocated to the latent class that exhibited the highest posterior probability of membership.

### Statistics

All analyses were carried out using R software v4.0.4 (https://www.r-project.org) and SPSS Statistics v25.0. Continuous variables were expressed as median with interquartile range (IQR), the distribution of which was assessed using the Kolmogorov-Smirnov tests. Mann-Whitney *U* tests were used to compare variables with a non-normal distribution and the Chi-squared tests were used for categorical variables between different latent classes. Propensity score matching (PSM) was applied for adjusting potential confounders using “MatchIt” R package with the nearest neighbor algorithm as matching method at a caliper value of 0.02 and a matching ratio of 1:2.^24^ Survival probabilities were estimated using the Kaplan-Meier analysis with log-rank test for difference between groups. Cox regression after PSM was conducted to identify more reliable causal inferences. Martingale residuals were used for nonlinearity analysis. A restricted cubic spline (RCS) was used to model and visualize the relationship of continuous predictors with all-cause mortality in elderly patients with NPC.^25^ Two-tailed *P*-value < .05 was deemed statistically significant.

## Results

### Patient characteristics

**Figure 1.**
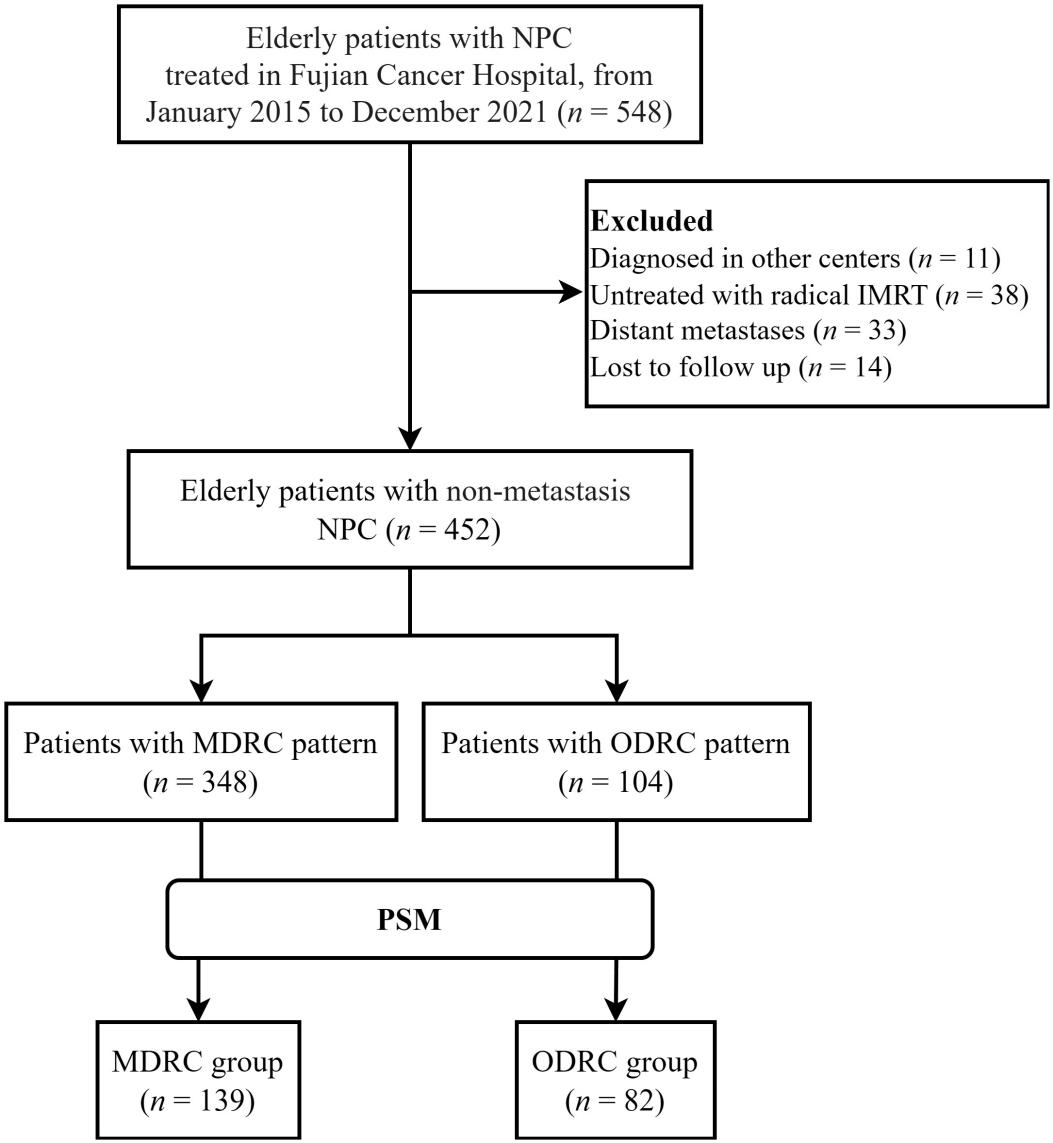
The flowchart of the patient selection. Abbreviations: IMRT, intensity-modulated radiotherapy; MDRC, metabolic disease-related comorbidity; NPC, nasopharyngeal carcinoma; ODRC, organ disease-related comorbidity; PSM, propensity score matching.

Among 548 elderly patients with NPC treated in our center, we excluded 36 cases discontinued or untreated with IMRT, 2 cases received palliative IMRT, 11 cases diagnosed in other centers, 33 cases presented distant metastases, and 14 cases lost to follow-up, 452 patients were included in the final study. The flowchart for the inclusion of patients is shown in Figure 1. The median follow-up time on the final follow-up date (conducted until October 31, 2023) was 34 months (IQR 19.5-56.1 months). During the follow-up, 114 patients (25.22%) died, 145 cases (32.08%) experienced disease progression, including 33 cases of locoregional recurrence (7.30%), and 37 cases of distant metastasis (8.19%). The rates of 3-year OS, LRFS, DMFS, and failure-free survival were 79.73%, 74.77%, 75.68%, and 71.86%, respectively.

### The prevalence and relevance of comorbidity

**Figure 2.**
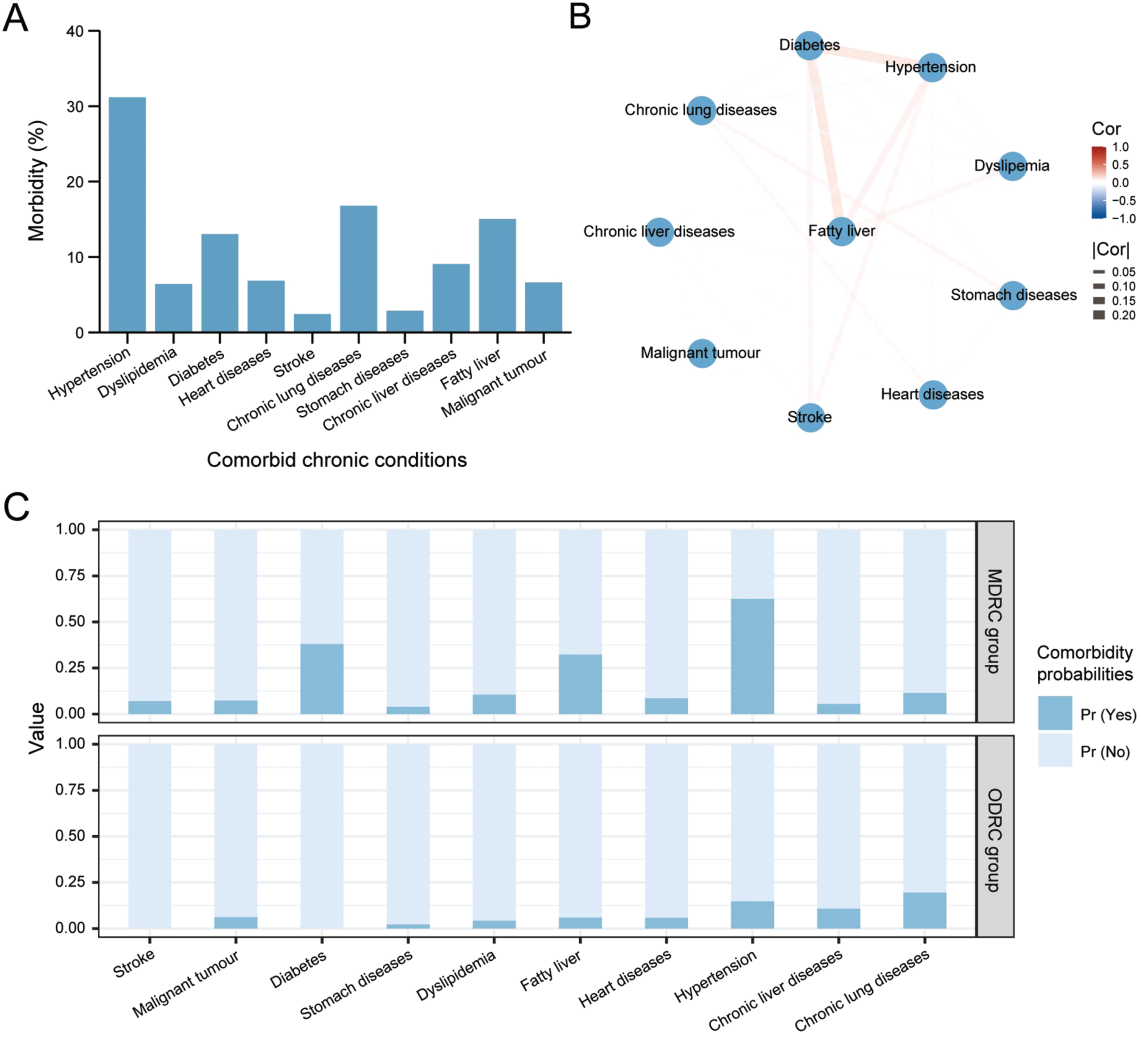
The comorbidity in elderly patients with NPC. (A) The prevalence of comorbid chronic conditions; (B) disease-disease interaction network of 10 broad disease classes according to the data of chronic diseases in the Chinese elderly. The thickness of lines is proportional to the disease co-occurrence; (C) item response probabilities among elderly patients with NPC across comorbidity patterns. Abbreviations: Cor, correlation; MDRC, metabolic disease-related comorbidity; NPC, nasopharyngeal carcinoma; ODRC, organ disease-related comorbidity; Pr, probability.

The overall prevalence of comorbidity in elderly patients with NPC was 65.93%. Figure 2A shows the morbidity of 10 chronic health conditions considered here. Hypertension had the highest prevalence (31.19%) at diagnosis in disease clusters, followed by chronic lung diseases (16.81%), fatty liver (15.04%), and diabetes (13.05%). The correlations among coexisting diseases were visualized by the web graph analysis. The hypertension-diabetes-fatty liver triad was observed to be the most powerful link, as shown in Figure 2B. Beyond that, the remaining combinations exhibited relatively weak correlations, indicating that the higher the prevalence of a specific chronic ailment, the greater its potential contribution to the occurrence of comorbid patterns.

### Identification of comorbidity patterns

In order to investigate the comorbidity patterns of elderly individuals with NPC, we conducted LCA by employing an iterative modeling approach. In each iteration, we introduced an additional class to generate a range of 2-5 latent class models. Subsequently, we assessed the relative goodness of fit among these models and determined the optimal number of latent classes. The model comprising 2 latent classes exhibited the most favorable fit, as evidenced by the lowest AIC and BIC values (Table 1). The class-conditional probabilities for each disease in 2 latent classes are presented in Figure 2C. In the 2-class solution model, hypertension, diabetes, fatty liver, and dyslipidemia seemed to be prevalent in class 1 that we labeled it as “metabolic disease-related comorbidity (MDRC)” class (*n* = 348, 76.99%). Another class was characterized by having low proportions overall, dominated by visceral diseases such as chronic lung and liver diseases, labeled as “organ disease-related comorbidity (ODRC)” group (*n* = 104, 23.01%). We also observed diabetes and stroke were only prevalent in the MDRC group. Hypertension exhibited prominent class-conditional probabilities in both groups, since its highest prevalence within our dataset. Moreover, heart diseases, stomach diseases, and malignant tumor feature low class-conditional probabilities in both classes, confirming the weak association between these diseases.

**Table 1.** Fit statistics for latent classes to identify an optimal model.

Latent class	AIC	BIC	G^2^	Chi-square
2	2833.230	2919.618	185.252	1796.401
3	2836.637	2968.275	166.658	1548.254
4	2839.158	3016.047	147.180	661.028
5	2845.877	3068.016	131.898	234.786

Abbreviations: AIC, Akaike information criterion; BIC, Bayesian Information Criterion; G^2^, likelihood ratio/deviance statistic.

### Individual oncological status in comorbidity patterns

The clinical features of the identified classes are presented in Table 2. Compared with patients in ODRC group, patients in MDRC group were more likely to be younger age at diagnosis (68 vs 69, *P* = .029) with more advanced stage (T stage, 73.85% vs 60.58%, *P* = .009; clinical stage, 87.07% vs 73.08%, *P* = .001). There was no statistically significant difference in gender, N stage, pretreatment plasma EBV DNA load, or treatment modality between the 2 groups. To mitigate potential confounding factors while preserving the sample size and information, patients were matched using PSM with replacement in this study to balance baseline profiles (Figure 1).^26^ A total of 139 patients in MDRC group and 82 in ODRC group were matched ultimately. No significant difference was found after PSM, suggesting better comparability between 2 classes.

**Table 2.** Baseline characteristics of patients with NPC before and after PSM matching, stratified by comorbidity patterns.

Characteristic	Before matching			After matching		
	MDRC group *n* = 348	ODRC group *n* = 104	*P*-value	MDRC group *n* = 139	ODRC group *n* = 82	*P*-value
Age (years)^a^	68 (65-71)	69 (66-73)	**0.029**	68 (66-71)	68 (66-71)	0.706
Gender			0.409			0.650
Male	268 (77.01)	76 (73.08)		103 (74.10)	63 (76.83)	
Female	80 (22.99)	28 (26.92)		36 (25.90)	19 (23.17)	
T stage			**0.009**			0.055
T1-2	91 (26.15)	41 (39.42)		31 (22.30)	28 (34.15)	
T3-4	257 (73.85)	63 (60.58)		108 (77.70)	54 (65.85)	
N stage			0.214			0.862
N0-1	197 (56.61)	66 (63.46)		78 (56.12)	47 (57.32)	
N2-3	151 (43.39)	38 (36.54)		61 (43.88)	35 (42.68)	
Stage			**0.001**			0.102
I-II	45 (12.93)	28 (26.92)		16 (11.51)	16 (19.51)	
III-IV	303 (87.07)	76 (73.08)		123 (88.49)	66 (80.49)	
EBV DNA_pre_^b^			0.156			0.868
<2000 copies/mL	197 (56.61)	67 (64.42)		88 (63.31)	51 (62.20)	
≥2000 copies/mL	151 (43.39)	37 (35.58)		51 (36.69)	31 (37.80)	
Treatment modality			0.058			0.435
RT alone	59 (16.95)	28 (26.92)		29 (20.86)	14 (17.07)	
IC + RT	122 (35.06)	34 (32.69)		46 (33.09)	25 (30.49)	
CCRT	33 (9.48)	13 (12.50)		11 (7.91)	12 (14.63)	
IC + CCRT	134 (38.51)	29 (27.88)		53 (38.13)	31 (37.80)	

Statistically significant results (*P* < 0.05) are shown in bold.

^a^Variables with non-normal distribution were represented as median with interquartile range.

^b^Based on previous studies in elderly patients with NPC,^16^ the cutoff level of pretreatment EBV DNA chosen was 2000 copies/mL in this study.

Abbreviations: CCRT, concurrent chemoradiotherapy; EBV DNA_pre_, pretreatment Epstein-Barr virus level; IC, induction chemotherapy; MDRC, metabolic disease-related comorbidity; NPC, nasopharyngeal carcinoma; ODRC, organ disease-related comorbidity; PSM, propensity score matching; RT, radiotherapy.

**Figure 3.**
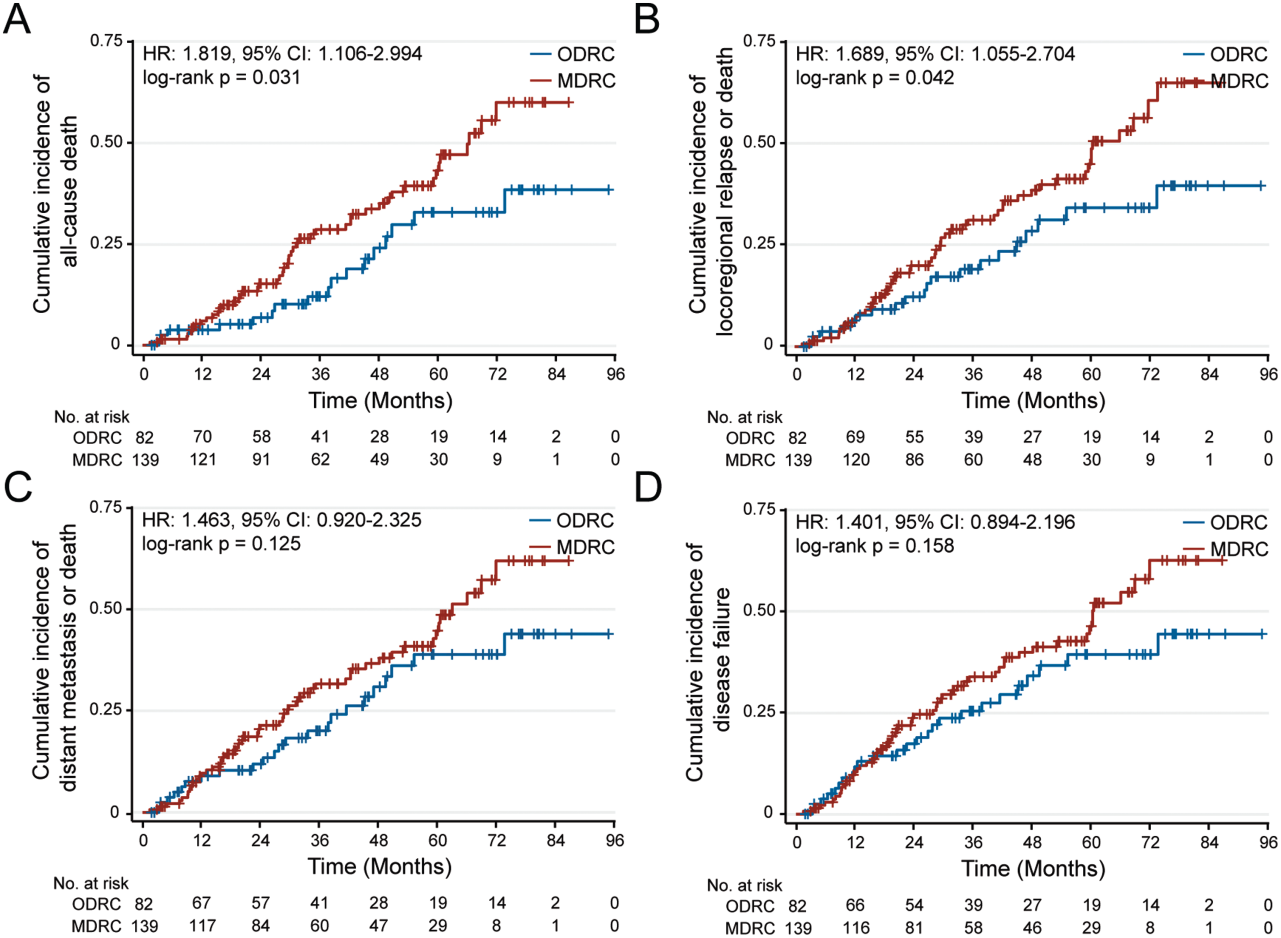
Kaplan-Meier failure estimates in elderly patients with NPC stratified by the comorbid groups. (A) Cumulative incidence of all-cause death; (B) cumulative incidence of locoregional failure or death; (C) cumulative incidence of distant metastasis or death; (D) cumulative incidence of disease failure. Abbreviations: MDRC, metabolic disease-related comorbidity; NPC, nasopharyngeal carcinoma; ODRC, organ disease-related comorbidity.

The 3-year OS of patients in MDRC and ODRC group was approximately 71.41% and 87.97%, respectively (HR 1.819 [95% CI, 1.106-2.994], *P* = .031, Figure 3A), and that of LRFS was approximately 68.73% and 80.88%, respectively (HR 1.689 [95% CI, 1.055-2.704], *P* = .042, Figure 3B), with significant differences. The 3-year DMFS in 2 groups was approximately 68.21% and 79.82%, respectively (HR 1.463 [95% CI, 0.920-2.325], *P* = .125, Figure 3C), whereas the 3-year failure-free survival was approximately 66.08%and 74.54%, respectively (HR 1.401 [95% CI, 0.894-2.196], *P* = .158, Figure 3D). Subgroup analysis suggested that patients with advanced stage in MDRC group showed a worse prognosis than those with early stage (67.33% in T3-4 vs 84.42% in T1-2, HR 2.438 [95% CI, 1.310-4.537], *P* = .023; 69.01% in 86.54% in stages III-IV vs stage I-II, HR 3.095 [95% CI, 1.460-6.561], *P* = .044), which was not observed in the ODRC group (84.57% in T3-4 vs 95.00% in T1-2, HR 2.785 [95% CI, 1.043-7.439], *P* = .092; 85.08% in stages III-IV vs 100.00% in stages I-II, HR 2.192 [95% CI, 0.704-6.829], *P* = .284, Supplementary Figure S1). Furthermore, there were no significant differences in the 3-year survival rates in the 2 comorbidity patterns for patients with different gender (71.00% in male vs 72.11% in female, HR 1.369 [95% CI, 0.744-2.519], *P* = .340 in MDRC group; 86.35% in male vs 92.86% in female, HR 1.377 [95% CI, 0.443-4.277], *P* = .613 in ODRC group, Supplementary Figure S1A), N stage (69.70% in N2-3 vs 72.82% in N0-1, HR 1.119 [95% CI, 0.634-1.976], *P* = .696 in MDRC group; 90.11% in N2-3 vs 85.87% in N0-1, HR 1.135 [95% CI, 0.436-2.958], *P* = .794 in ODRC group, Supplementary Figure S1C), and plasma EBV DNA load (61.63% in EBV DNA_pre_ ≥ 2000 copies/mL vs 78.42% in EBV DNA_pre_ < 2000 copies/mL, HR 1.437 [95% CI, 0.800-2.581], *P* = .205 in MDRC group; 85.66% in EBV DNA_pre_ ≥ 2000 copies/mL vs 88.98% in EBV DNA_pre_ < 2000 copies/mL, HR 1.096 [95% CI, 0.414-2.905], *P* = .852 in ODRC group, Supplementary Figure S1E). Finally, we analyzed the differences of survival benefits in 2 comorbidity patterns (Supplementary Figure. S1F). It confirmed that no significant differences were found between different treatment modes in MDRC group (*P* = .633), but there was in the ODRC group (*P* = .048). Further analysis showed that patients could benefit more from concurrent chemotherapy in ODRC group (HR 5.894 [95% CI, 2.264-15.355], *P* = .002, Supplementary Figure S1H), but not IC (HR 1.857 [95% CI, 0.639-5.392], *P* = .201, Supplementary Figure S1G).

### Influential factors for comorbidity patterns

**Figure 4.**
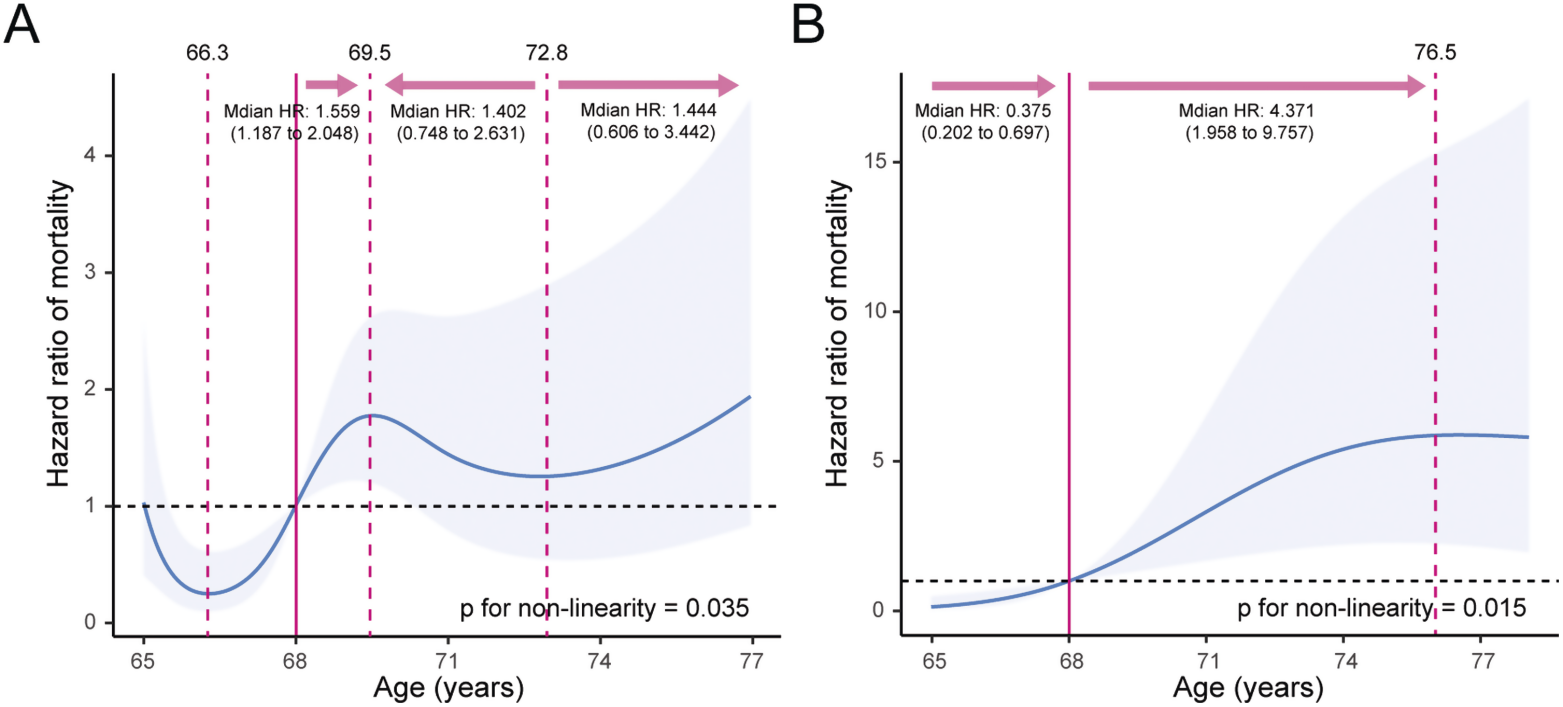
Restricted cubic spline models for the relationship between age and the risk of mortality in (A) MDRC group and (B) ODRC group. The curve and areas represent the estimated HRs and their 95% CIs. The vertical solid line represents the clinical reference age (HR = 1), and the dashed lines represent the point where the slope suddenly decreases the most, corresponding to the age. Abbreviations: MDRC, metabolic disease-related comorbidity; ODRC, organ disease-related comorbidity.

We used Cox regression analysis to identify prognostic factors. We found age was identified as independent prognostic factor in patients both in MDRC (HR 1.123 [95% CI, 1.041-1.212], *P* =.003) and ODRC group (HR 1.282 [95% CI, 1.119-1.468, *P* <.001], Supplementary Table S1), the risk of poor OS substantially increased with age. We tested the nonlinearity of age with mortality risk, as shown in Supplementary Figure S2. To further explore the relationship between age and mortality risk of patients in comorbidity patterns, Cox regression model with an age timescale was fitted for all-cause mortality using RCS (smooth curve). We used equally spaced knots and determined the appropriate number of knots by fitting models with varying knot quantities (ranging from 3 to 7), that yielded the lowest AIC, highest R^2^ or Somers’ *Dxy* rank correlation were selected as the optimal choice (5 knots in MDRC group and 3 knots in ODRC group, Table 3). We found the nonlinear relationships between age and mortality risk in MDRC group (*P* = .035, Figure 4A) and ODRC group (*P* = .015, Figure 4B). Moreover, the inflection points at the age of 68 for mortality risk can be both observed, when age over 68, the HR was significantly higher than 1. The age associated with the lowest risk of mortality was approximately 66 in the MDRC group. The value of HR has an initial steep increase when age ranges from 66 to 70, then plateaued. Each unit increase of age was associated with a 55.9% increase of the risk of mortality between age 68 and 70 (HR 1.559, 1.187-2.048). There was a trend for decreasing the risk of mortality when age was between 70 and 73, whereafter, the risk gradually rising with increasing age. Nevertheless, the risk of mortality up perpetually with age increasing in ODRC group, until which reached a plateau when the age was around 77. Above 68 years old, the HR per standard deviation higher predicted mortality was 4.371 (1.958-9.757).

**Table 3.** Fit statistics for RCS knot quantities to identify an optimal model in comorbidity patterns.

The number of RCS knots	MDRC group			ODRC group		
	AIC	*R* ^2^	Somers’ *Dxy* rank correlation	AIC	*R* ^2^	Somers’ *Dxy* rank correlation
3	399.134	0.074	0.236	110.197	0.277	0.711
4	400.797	0.076	0.276	112.111	0.278	0.711
5	393.446	0.140	0.378	112.522	0.297	0.696
6	395.389	0.140	0.369	114.246	0.300	0.711
7	398.049	0.136	0.378	111.779	0.352	0.719

Abbreviations: AIC, Akaike information criterion; MDRC, metabolic disease-related comorbidity; ODRC, organ disease-related comorbidity; RCS, restricted cubic spline.

## Discussion

In our cohort of elderly patients with NPC, a substantial prevalence of comorbid conditions was observed, with approximately two-thirds of the participants exhibiting comorbidity. A more detailed look at the combinations of diseases highlighted a diversity in the comorbid conditions observed. Specifically, we identified 2 comorbidity patterns, MDRC, characterized by conditions such as hypertension, diabetes, and fatty liver, and ODRC, dominated by visceral diseases. Notably, individuals with MDRC displayed a significantly heightened risk of both all-cause mortality and locoregional relapse. In addition, an intriguing inverted S-shaped relationship between age and all-cause mortality was discovered among patients aged 68 years and older, presenting with the MDRC pattern. Nevertheless, a higher age was found to be associated with a greater risk of mortality among patients presenting with the organ-related comorbidity pattern, specifically within the age range of 68-77 years.

The incidence and mortality rate of NPC exhibit a gradual increase with advancing age, and considering the rising aging population in China, the burden of this disease is poised to grow.^7^ Comorbidity has emerged as an important factor influencing treatment selection and survival outcomes in patients with NPC.^13,27^ Various scoring tools have been used to quantify underlying comorbidity in patients with NPC.^12,15^ Huang et al^13^ used CCI score to evaluate 1137 elderly patients with NPC who received definitive radiotherapy, and the comorbidity incidence was 22.4%. In endemic regions, it demonstrated a comorbidity prevalence of 42.2% using the ACE-27 by Guo et al,^28^ with gastrointestinal diseases proving most common. In non-endemic regions, the comorbidity incidence was 44%, with cardiovascular and pulmonary diseases predominating, which also used the ACE-27.^29^ Notably, these methods often presented in score form and lack detailed insights into individual comorbid diseases. In our study, the comorbidity rate of 65.93% surpassed that of other investigations, likely attributed to our comprehensive documentation of specific diseases for comorbidity assessment. Furthermore, we included common health conditions, such as hypertension, which also exhibited the highest comorbidity incidence in our cohort. Currently, the management of chronic diseases, such as diabetes and hypertension, has seen widespread implementation in China and has yielded certain outcomes.^30^ However, the current approach to chronic disease management primarily emphasizes the management of single disease paradigm.^30^ There remains a dearth of comprehensive management strategies for addressing comorbidities among the elderly cancer population, including those diagnosed with NPC.

Studies have shown that the association between chronic diseases is not accidental, and the high correlation of comorbidity has a certain distribution law.^31^ Indeed, the clinical efficacy of any intervention can be enhanced by identifying specific combinations that may necessitate an alternative diagnostic or therapeutic approach.^32^ Thus, unraveling the comorbidity pattern derived from the associative nature of comorbidities holds immense value for disease classification and serves as a reference for managing chronic illnesses in elderly patients with cancer. However, there lacks a consensus regarding universally recognized methods for identifying comorbidity patterns currently. Noteworthy techniques commonly used in research and clinical practice include factor analysis, cluster analysis, LCA, association rules, and network analysis.^32-35^ LCA presents a meticulous and astute approach for grouping categorical indicators, devoid of preconceived assumptions about associations or predetermined concepts, so that it focuses more on individual features, thereby minimizing confirmation biases.^21^ In this study, network analysis revealed potential associations between hypertension, diabetes, and fatty liver. Similar trends were identified in several other studies, indicating the most prevalent comorbidities alongside hypertension and diabetes in the elderly.^36,37^ We further used LCA to gain deeper insights into the comorbidity patterns. We identified disease combinations exhibiting high correlation among hypertension, diabetes, and fatty liver as the metabolic disease-related pattern, while the other group was referred to as the organ disease-related pattern. The lower incidence of organ-related diseases observed in our study cohort can likely be attributed to the fact that all cases originated from specialized oncology hospitals. Due to safety concerns, elderly patients with severe medical conditions may more frequently receive treatment at general hospitals. Previous studies have demonstrated that the aforementioned comorbidity pattern shares common risk factors and pathophysiology.^38^ Consequently, a more detailed analysis pertaining to concerning comorbidity among the elderly NPC population holds considerable potential in guiding treatment planning, preventive measures, and the formulation of comprehensive health care policies that encompass systemic considerations.

Gaining a comprehensive comprehension of comorbidity patterns and their management holds significant implications in the realm of cancer treatment.^32^ In terms of cancer prevention practices, individuals with comorbid hypertension or diabetes often maintain more frequent contact with medical services, resulting in an increased likelihood of early cancer detection.^39^ This may be the reason why cancer is diagnosed earlier in patients exhibiting metabolic disease-related pattern than in the other group in this study. Furthermore, within our cohort of patients encompassing both comorbidity patterns, individuals with a MDRC pattern demonstrated a higher risk of mortality. Numerous findings indicate that hypertension, diabetes, and obesity are associated with an elevated risk of developing various types of cancers.^40,41^ However, the causal link between these metabolic disorders and the risk of death from NPC remains unclear. As prevalent chronic metabolic disorders, the pathogenesis of these conditions is influenced by diverse factors.^42^ The metabolic aberrations possess the potential to significantly contribute to cancer development and progression through the regulation of distinct signaling pathways.^43^ For instance, metabolic disturbances such as dyslipidemia, hyperinsulinemia, and hyperglycemia in diabetes may increase the risk, hasten the progression, and heighten mortality rates of cancers.^41^ Consequently, numerous studies are currently exploring the potential anticancer properties of antidiabetic medications.^29^ Furthermore, our study revealed that patients exhibiting a metabolic disease-related pattern displayed more unfavorable prognosis in the advanced stage. It is worth noting that each group comprised only 16 patients with early stage. Consequently, it should be cautious when extrapolating these findings to the overall stage. Concurrent chemoradiotherapy (CCRT) has been widely accepted as a standard treatment modality for locally advanced NPC.^12^ Nevertheless, the benefit of chemotherapy for elderly patients with NPC remains controversial.^12^ While Sommat et al observed that the addition of chemotherapy was not associated with improved survival, a study based on the Surveillance, Epidemiology, and End Results database indicated that CCRT treatment yielded longer OS compared to radiotherapy alone in patients with NPC.^27,44^ In our present investigation, we identified that patients with ODRC pattern may derive greater benefit from concurrent chemotherapy. As we all know, the addition of chemotherapy is linked to elevated risks of acute and late toxicity.^45^ Consequently, the application of chemotherapy in elderly NPC individuals should be weighed carefully.

In addition to comorbid status, another characteristic that should be taken into account in the management of elderly patients with NPC is age.^46^ Age was considered as an important role in the survival outcome of the elderly patients with NPC, as expected, older age has independently been associated with poorer OS and an increased risk of mortality.^9,47^ In this study, age was also proved to have a negative influence on OS in both MDRC group and ODRC group by the multivariable analysis. This highlighted the significance of early detection and treatment in this population. Rather than employing a fixed cutoff value such as 75 or 85 to classify elderly patients into young old, older old, and oldest old, we observed a gradually escalating prevalence of mortality with increasing age until reaching a plateau at the age of 77 among patients with the ODRC pattern.^48^ While in patients with MDRC, there was a trend for increasing the risk of mortality between the age of 66-70 and over 73. Accordingly, both comorbid conditions and age should be taken into account to make the most appropriate management decisions for each patient.

To the best of our knowledge, there is a paucity of research delving into the impact of comorbidity patterns on mortality risk among elderly patients with NPC. The primary strength of this investigation lies in the comprehensive exploration of associations between NPC prognosis and comorbidities based on clinical comorbid information from real-world case records. In spite of the valuable implication it presents, it is essential to acknowledge one major limitation related to the utilization of baseline disease information, which may change over the course of the follow-up period. However, a prior study has demonstrated relative consistency in comorbidity patterns between baseline assessment and the second resurvey.^49^ Participants with severe diseases may have succumbed to mortality prior to the survey, thereby implying that the identified patterns likely reflect comorbidity profiles among long-term survivors. Another limitation of this study pertains to the somewhat limited number of diseases included in the baseline survey, despite the incorporation of common multisystemic conditions. Given the absence of standard exists for measuring multimorbidity, the selection of specific morbidities to examine is inherently subjective and depends on the data available.^50^ For instance, due to the focus on cancer screening during baseline assessments, an inherent bias toward under-reporting of undiagnosed conditions, such as prevalent chronic ailments like visual or hearing impairments among the elderly, may be present.^33^ Furthermore, despite employing PSM to mitigate the influence of initial confounding factors, there was still an inevitable potential for bias subsequent to PSM (for instance, the *P*-value pertaining to T stage in MRDC and ORDC group (.055) remained in close proximity to the boundary of statistical significance). Additionally, the median follow-up time in the current survey was relatively limited, and comprehensive data regarding the duration and severity of chronic diseases were not captured. Extending the follow-up duration in future studies or further prospective cohort studies could facilitate a more comprehensive understanding of the complex interplay between comorbidity patterns and relevant factors, corroborating and supplementing the findings.

## Conclusion

In summary, our investigation unveils potential comorbidity patterns among elderly patients with NPC. An enhanced integrative multidisciplinary strategy on enhanced management and prevention strategies for these conditions holds promising potential for enhancing the prognosis of elderly patients with NPC.

## Supplementary Material

oyae063_suppl_Supplementary_Figures_S1-S2

oyae063_suppl_Supplementary_Tables_S1

## Data Availability

The data underlying this article cannot be shared publicly due to the privacy of individuals who participated in the study. The data will be shared on reasonable request to the corresponding author.
