# Supplementary material for: Comorbidity profiling identifies potential subtype of elderly patients with nasopharyngeal carcinoma
Source: Oncologist. 2024 Apr 16;29(8):e1020–30. doi: 10.1093/oncolo/oyae063 (PMC11299953; doi:10.1093/oncolo/oyae063)
Supplement: oyae063_suppl_Supplementary_Figures_S1-S2 [file oyae063_suppl_supplementary_figures_s1-s2.docx]

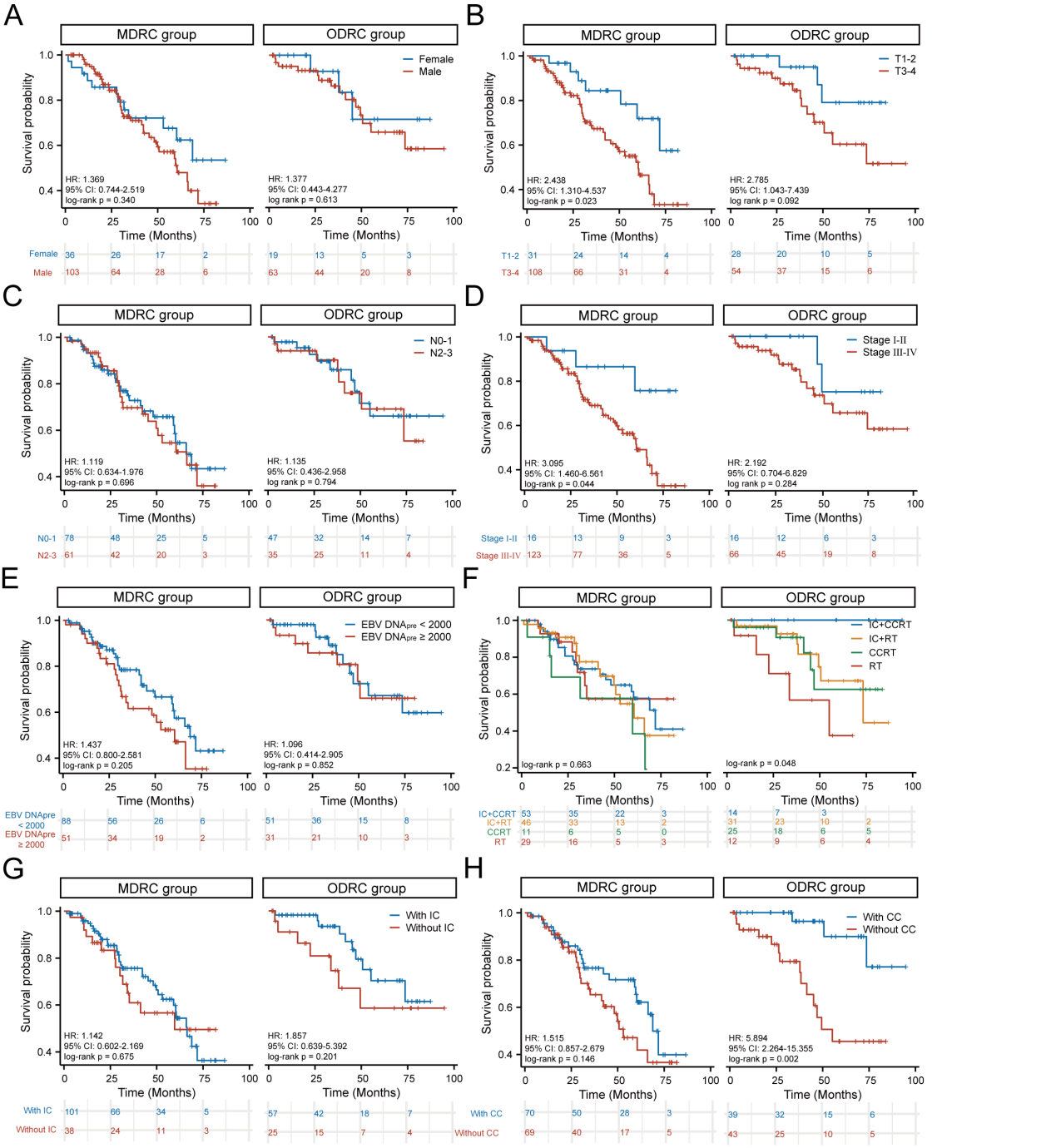


**Supplemental Figure S1.** Survival analysis of overall survival for elderly NPC patients at different comorbidity patterns with different characteristics: (**A**) gender; (**B**) T stage; (**C**) N stage; (**D**) clinical stage; (**E**) pre-treatment plasma EBV DNA load; (**F**) treatment modality; (**G**) the application of induction chemotherapy; (**H**) the application of concurrent chemotherapy. CC, concurrent chemothrapy; CCRT: concurrent chemoradiothrapy; EBV DNA_pre_: pre-treatment Epstein-Barr virus level; IC: induction chemotherapy; MDRC: metabolic disease-related comorbidity; NPC: nasopharyngeal carcinoma; ODRC: organ disease-related comorbidity; RT: radiotherapy.


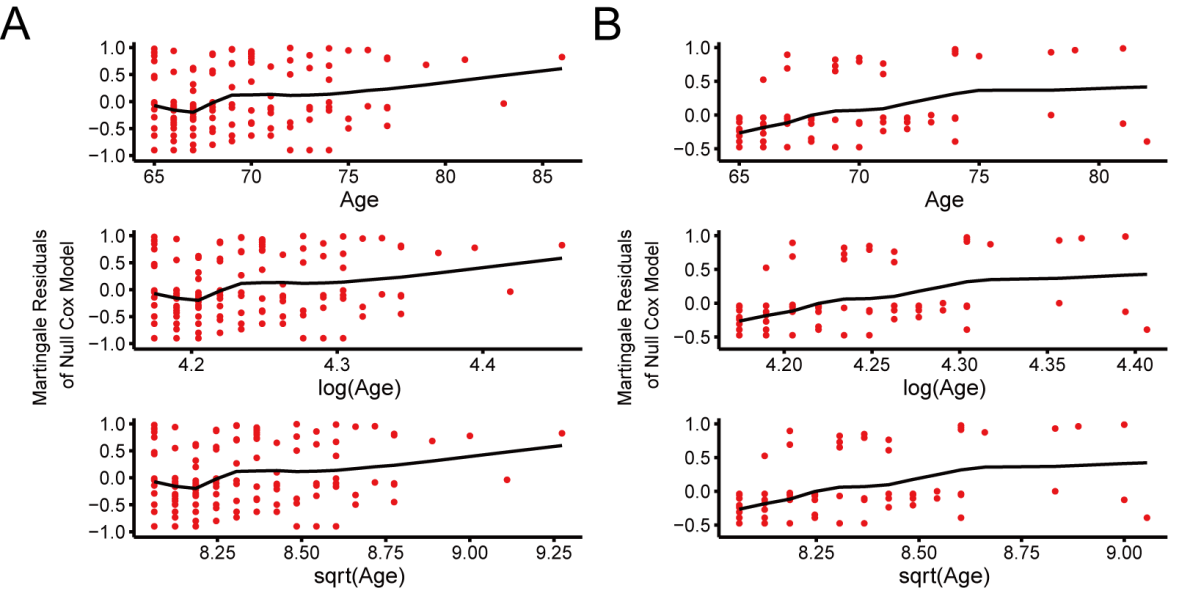


**Supplemental Figure S2.** Association between age and the risk of mortality in comorbidity patterns assessed by Martingale residuals, allowing for nonlinear effects: (**A**) MDRC group; (**B**) ODRC group. MDRC: metabolic disease-related comorbidity; ODRC: organ disease-related comorbidity.
