# Supplementary material for: Comorbidity profiling identifies potential subtype of elderly patients with nasopharyngeal carcinoma
Source: Oncologist. 2024 Apr 16;29(8):e1020–30. doi: 10.1093/oncolo/oyae063 (PMC11299953; doi:10.1093/oncolo/oyae063)
Supplement: oyae063_suppl_Supplementary_Tables_S1 [file oyae063_suppl_supplementary_tables_s1.docx]

| **Supplemental Table S1.** Cox regression analysis of prognostic factors after PSM in elderly NPC patients with different comorbidity patterns. | | | | | | | | |
| --- | --- | --- | --- | --- | --- | --- | --- | --- |
| Comorbidity patterns | Variables | Group | Univariate analysis | |  | Multivariate analysis | |  |
|  |  |  | HR (95% CI) | *P* value |  | HR (95% CI) | *P* value |  |
| MDRC  group | Age (years) | continuous | 1.118 (1.049-1.193) | **<0.001** |  | 1.123 (1.041-1.212) | **0.003** |  |
|  | Gender | Male vs. Female | 1.376 (0.713-2.659) | 0.342 |  | 1.697 (0.833-3.458) | 0.145 |  |
|  | T stage | T3-4 vs. T1-2 | 2.480 (1.106-5.562) | **0.027** |  | 1.630 (0.544-4.885) | 0.383 |  |
|  | N stage | N2-3 vs. N0-1 | 1.119 (0.635-1.973) | 0.696 |  | 0.911 (0.474-1.752) | 0.781 |  |
|  | Stage | Stage Ⅲ-Ⅳ vs. Stage Ⅰ-Ⅱ | 3.145 (0.972-10.171) | 0.056 |  | 1.686 (0.306-9.287) | 0.549 |  |
|  | EBV DNA_pre_ (copies/mL)^a^ | ≥ 2000 vs. <2000 | 1.443 (0.814-2.558) | 0.209 |  | 1.320 (0.721-2.416) | 0.368 |  |
|  | Treatment modality | RT vs. CCRT vs. IC+RT vs. IC+CCRT | 0.938 (0.728-1.209) | 0.621 |  | 1.059 (0.764-1.468) | 0.731 |  |
|  |  |  |  |  |  |  |  |  |
| ODRC  group | Age (years) | continuous | 1.182 (1.092-1.280) | **<0.001** |  | 1.282 (1.119-1.468) | **<0.001** |  |
|  | Gender | Male vs. Female | 1.378 (0.396-4.804) | 0.614 |  | 1.097 (0.291-4.138) | 0.892 |  |
|  | T stage | T3-4 vs. T1-2 | 2.797 (0.802-9.753) | 0.106 |  | 3.174 (0.340-29.621) | 0.311 |  |
|  | N stage | N2-3 vs. N0-1 | 1.135 (0.438-2.946) | 0.794 |  | 1.842 (0.480-7.068) | 0.374 |  |
|  | Stage | Stage Ⅲ-Ⅳ vs. Stage Ⅰ-Ⅱ | 2.197 (0.502-9.624) | 0.296 |  | 0.534 (0.031-9.239) | 0.666 |  |
|  | EBV DNA_pre_ (copies/mL)^a^ | ≥ 2000 vs. <2000 | 1.097 (0.416-2.892) | 0.852 |  | 0.419 (0.113-1.553) | 0.193 |  |
|  | Treatment modality | RT vs. CCRT vs. IC+RT vs. IC+CCRT | 0.863 (0.552-1.350) | 0.519 |  | 1.394 (0.708-2.744) | 0.337 |  |
| Statistically signifcant results (*P* < 0.05) are shown in bold.  ^a^: Based on previous studies in elderly patients with NPC^16^, the cutoff level of pretreatment EBV DNA chosen were 2000 copies/ml in this study. Abbreviations: CCRT: concurrent chemoradiothrapy; EBV DNA_pre_: pre-treatment Epstein-Barr virus level; IC: induction chemotherapy; MDRC: metabolic disease-related comorbidity; NPC: nasopharyngeal carcinoma; ODRC: organ disease-related comorbidity; PSM: propensity score matching; RT: radiotherapy. | | | | | | | | |
